# Asynchronous Remote Assessment for Cognitive Impairment: Reliability Verification of the Neurotrack Cognitive Battery

**DOI:** 10.2196/34237

**Published:** 2022-02-18

**Authors:** Jennifer Rae Myers, Jordan M Glenn, Erica N Madero, John Anderson, Rachel Mak-McCully, Michelle Gray, Joshua L Gills, John E Harrison

**Affiliations:** 1 Neurotrack Technologies Inc Redwood City, CA United States; 2 University of Arkansas Fayetteville, AR United States; 3 Metis Cognition Ltd Kilmington Common United Kingdom; 4 Alzheimer Center VU Medical Center Vrije Universiteit Amsterdam Amsterdam Netherlands; 5 Institute of Psychiatry, Psychology & Neuroscience King's College London United Kingdom

**Keywords:** cognition, screening, remote testing, psychometric, challenge, validation, assessment, impairment, access, reliability, stability, testing, utility

## Abstract

**Background:**

As evidenced by the further reduction in access to testing during the COVID-19 pandemic, there is an urgent, growing need for remote cognitive assessment for individuals with cognitive impairment. The Neurotrack Cognitive Battery (NCB), our response to this need, was evaluated for its temporal reliability and stability as part of ongoing validation testing.

**Objective:**

The aim of this study is to assess the temporal reliability of the NCB tests (5 total) across a 1-week period and to determine the temporal stability of these measures across 3 consecutive administrations in a single day.

**Methods:**

For test-retest reliability, a range of 29-66 cognitively healthy participants (ages 18-68 years) completed each cognitive assessment twice, 1 week apart. In a separate study, temporal stability was assessed using data collected from 31 different cognitively healthy participants at 3 consecutive timepoints in a single day.

**Results:**

Correlations for the assessments were between 0.72 and 0.83, exceeding the standard acceptable threshold of 0.70 for temporal reliability. Intraclass correlations ranged from 0.60 to 0.84, indicating moderate to good temporal stability.

**Conclusions:**

These results highlight the NCB as a brief, easy-to-administer, and reliable assessment for remote cognitive testing. Additional validation research is underway to determine the full magnitude of the clinical utility of the NCB.

## Introduction

### Background

Remote cognitive assessment, through the use of digital tools, represents an efficient means for individuals to assess their levels of function, without needing to visit an in-person clinic. These tools allow for the “benchmarking” of current levels of function, as well as the detection of change over time. Monitoring changes in performance over time enables the detection of progressive decline that might be indicative of neurological disease. It also enables the detection of improvement from interventions targeting lifestyle changes and modifiable risk factors aimed at improving cognitive health [1-3]. Remote assessment also plays a role in areas such as screening for clinical trial participation, postmarketing surveillance of licensed therapies, and the collection of data in large prospectively ascertained cohorts. Clinical research has yielded a number of candidate measures for indexing key cognitive skills, with experts largely agreeing that, in studies of individuals living with Alzheimer disease, assessments should measure attention, memory, and executive function [4,5].

### Development of the Neurotrack Cognitive Battery

Although there are other brief and computerized cognitive assessments available, such as the Cambridge Neuropsychological Test Automated Battery (CANTAB), Savonix, and BrainCheck, the Neurotrack Cognitive Battery (NCB) offers several distinct platform features to address common concerns associated with cognitive and remote assessment. These include web camera capability, objective scoring via algorithms, and the ability to be administered without the need for a trained health care professional. Regarding assessment design, there are several recommended properties of an ideal cognitive assessment tool, which includes the need to assess the full range of relevant cognitive processes, be sensitive to aging and cognitive deficits, contain equivalent versions for repeat administration, have a reasonable testing duration, and have good reliability and validity metrics [6]. The application of recommended test selection properties led us to develop digital versions of Part B of the Trail Making Test [7] as a measure of executive function, as well as a computerized novel variant of the Digit Symbol Substitution (DSST) paradigm. The DSST enjoys the virtues of brevity and reliability, as well as being a well-known general measure of cognitive function that is sensitive to subtle changes in cognition [8]. To further index these functions, we selected the Erikson flanker task and the go/no-go test [9]. To extend the coverage of the assessment to include episodic memory, we also included a paired associate learning task in which individuals are required to pair shopping list items with the associated prices. These assessments were combined with a previously validated associative and recognition memory visual paired comparison paradigm [10]. This task is based on research conducted by Zola et al [11] and utilizes eye tracking to determine novelty preference as an index of memory.

Measures of cognitive function must be reliable, sensitive, and valid to show meaningful change over time [12]. In the early stages of test development, we have focused on ensuring the scientific validity of the Neurotrack assessments through standard psychometric testing. Thus, the aims of our research were to (1) assess the temporal reliability of the NCB tests across a 1-week period and (2) determine the temporal stability of these measures across 3 consecutive administrations in a single day.

## Methods

### Participants

#### Temporal Reliability

Participants were workers recruited through Amazon Mechanical Turk (MTurk) and Prolific, crowdsourcing websites used for research recruitment and testing. The use of MTurk and Prolific has shown to be comparable to traditional research methods and allow for greater access to hard-to-reach and diverse populations [13,14]. Up to 150 subjectively cognitively healthy participants in the United States were recruited for each assessment separately. Individuals who successfully completed an assessment at time point 1 were granted access to retake the assessment 1 week later at time point 2. Participants were compensated up to US $2.85 for each assessment completed. Participant characteristics for each assessment are outlined in Table 1.

**Table 1:** Participant characteristics for temporal reliability.

Characteristics			NCB^a^ assessment and participants								
			Path points, n=66		Symbol match, n=29		Arrow match, n=46		Light reaction, n=46		Item price, n=51
Age (years), mean (SD)			32.82 (7.56)		28.97 (9.08)		33.39 (7.73)		39.50 (11.34)		36.53 (10.18)
**Sex, n (%)**											
	Female	15 (23)		12 (41)		11 (24)		19 (41)		14 (27)	
	Male	51 (77)		17 (59)		35 (76)		27 (59)		37 (73)	
**Race, n (%)**											
	Nonwhite	12 (18)		8 (28)		11 (24)		8 (17)		11 (22)	
	White	54 (82)		21 (72)		35 (76)		38 (83)		40 (78)	

^a^NCB: Neurotrack Cognitive Battery.

#### Temporal Stability

Potential participants were recruited through a research interest listserv and by word of mouth; these individuals were selected from a different pool than the original group, with no overlap present between the two. A total of 55 individuals who were subjectively cognitively healthy expressed interest in participating in the study. Of the 55 individuals, 31 completed the study in its entirety. The mean age of the participants was 51 (SD 17.61) years. Regarding other participant characteristics, 41 of the 55 participants (75%) had a college degree, 41 (75%) were female, and 21 (38%) identified as a person of color. Participants were asked to complete the entire NCB 3 consecutive times in a single day, which took approximately 60 minutes. Participants who completed the study received a US $50 electronic gift card.

### Measures and Procedure

As previously mentioned, the following measures were selected for use based on their relative ease of administration, brevity, and tendency to represent reliable measures of cognition:

- Path points is a 2-minute assessment of executive function. This task requires participants to connect dots alternating between a number and a letter in ascending order. The primary assessment score is based on completion time.
- Symbol match is a 2-minute assessment of processing speed. Participants are instructed to determine whether 2 symbols are equal or unequal based on a legend with 9 number/symbol pairs. Participants must complete as many trials as they can in 2 minutes. Primary scores are based on accuracy and speed.
- Arrow match is a 3-minute assessment of attention. This task requires participants to indicate which direction the center arrow is pointing (left or right) among 4 distractor arrows. Primary scores are based on accuracy and speed.
- Light reaction is a 3-minute assessment of inhibition. This task requires participants to respond when they see a green light and refrain from responding when they see a red light. The primary assessment score is based on accuracy and speed.
- Item price is a 3-minute assessment of associative learning. This task requires participants to learn the prices of various produce items (eg, bananas, carrots) and identify the correct price during the recognition trials. Primary scores are based on accuracy.

For temporal reliability and stability, data were collected separately, using new participants for each, in an unsupervised remote setting. Participants were instructed to complete the assessments when feeling rested and in a private room to optimize focus. Participants were also instructed to complete the assessments using a laptop or desktop computer with a physical keyboard, mouse, and camera, with their device charged to at least 20%, and on a stable internet connection. Study protocols were approved by the University of Arkansas Institutional Review Board and participants provided informed consent prior to study enrollment.

## Results

### Temporal Reliability

**Figure 1:**
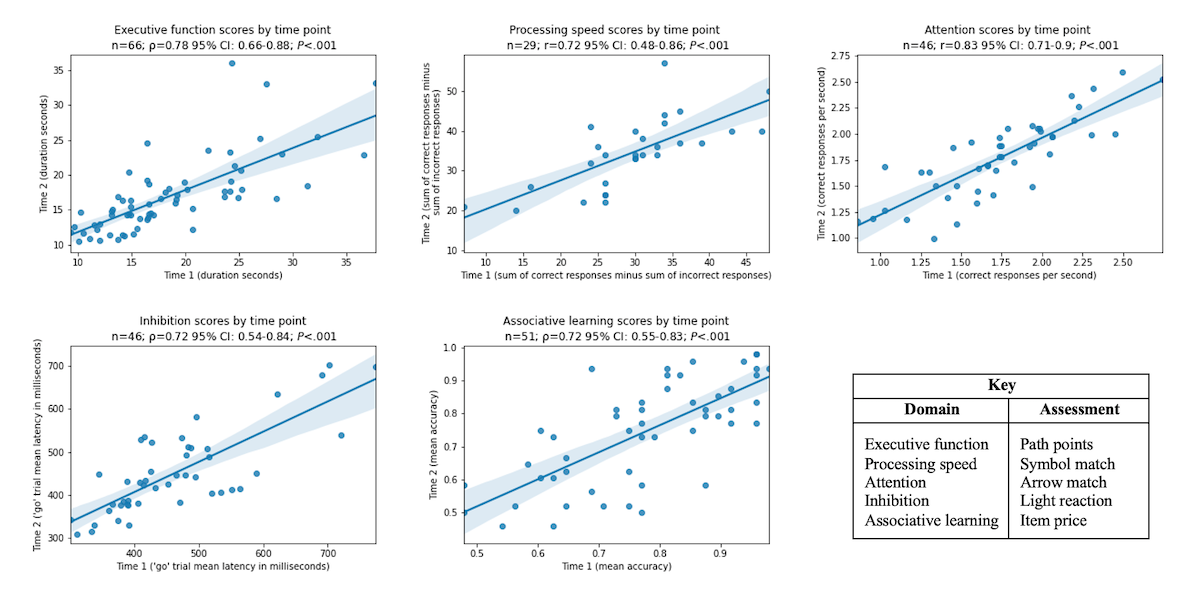
Scatterplots of test-retest scores for the Neurotrack Cognitive Battery (NCB) assessments. The Pearson correlation coefficient is represented by r. The Spearman correlation coefficient represented by the Greek letter &rho;.

For each assessment, the test-retest reliability of the scores from time point 1 and 2 were assessed using both Pearson and Spearman correlations (Figure 1). In instances where a visual inspection of the data suggested a general monotonic relationship, the Spearman correlation coefficient was selected. Outliers, defined as scores more or less than 5 standard deviations from the mean, were removed from the final analyses. Correlations for the assessments ranged from 0.72 to 0.83, indicating acceptable to good test-retest reliability of the NCB.

### Temporal Stability

Temporal stability was examined by calculating estimates of within-subject standard deviation (*s*_w_) and intraclass correlation coefficients (ICCs) for each assessment. The *s*_w_ is used to quantify measurement error in repeated measurements as a single overall measure. Results are outlined in Table 2.

**Table 2:** Within-subject mean and standard deviation (s_w_) and Kendall rank correlation coefficient (Kendall τ) values for Neurotrack Cognitive Battery (NCB) assessments (n=31 participants).

NCB assessment	Within-subject mean	*s*_w_	Kendall’s τ	95% CI
Path points^a^	13.47	0.27	–0.42	2.48-3.57
Symbol match^b^	29.80	4.32	–0.02	3.54-5.10
Arrow match^c^	1.51	0.15	0.15	0.12-0.17
Light reaction^d^	476.69	52.25	–0.11	42.53-61.96
Item price^e^	0.74	0.08	–0.20	0.07-1.00

^a^Score is measured in seconds.

^b^Score is the sum of correct response minus the sum of incorrect responses.

^c^Score is the number of correct responses per second.

^d^Score is measured in milliseconds.

^e^Score is the mean accuracy of responses.

**Figure 2:**
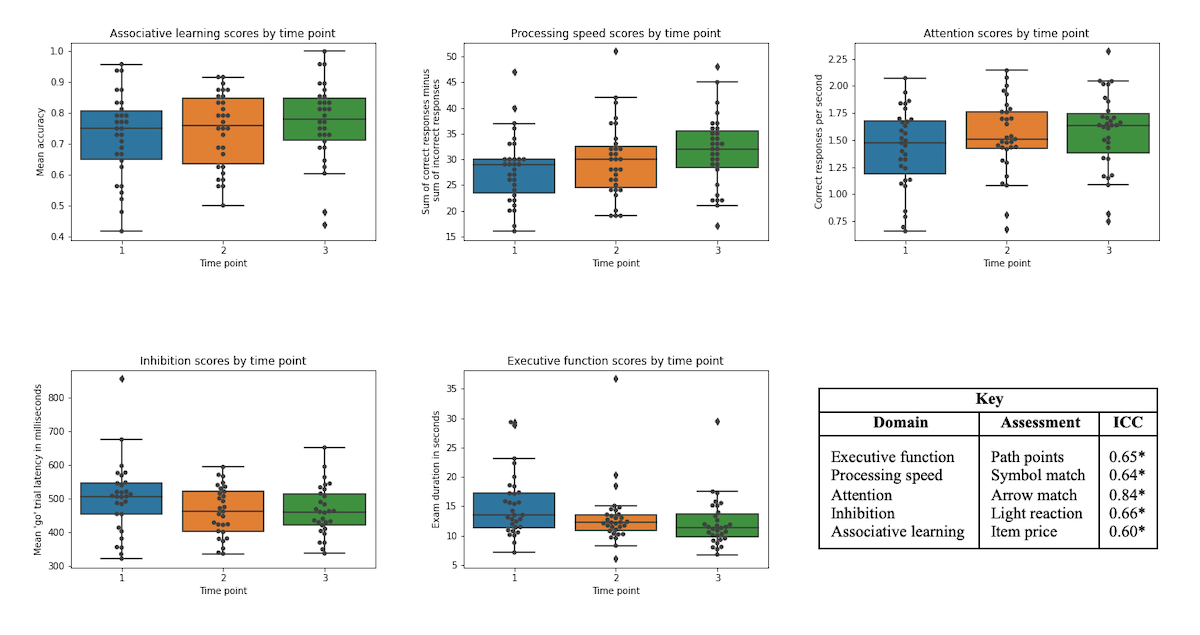
Boxplots representing scores from each assessment separated by time point and intraclass correlation coefficients (ICCs). The asterisk indicates *P*<.001. n=31 participants.

It should be noted that *s*_w_ is based on the assumption that the *s*_w_ is independent from the within-subject mean (assessed using the Kendall rank correlation coefficient [Kendall τ]). Thus, for the path points (executive function) and item price (associative learning) assessments, there is the possibility of overestimation or underestimation in how close scores are to the mean. ICCs were also calculated to assess variation due to measurement error. The ICC (2,1) was selected as it is the recommended ICC form to use for test-retest metrics [15,16]. Correlations for the assessments ranged from 0.60 to 0.84, indicating moderate to good reliability. Boxplots for each assessment are depicted in Figure 2.

## Discussion

### Principal Findings

The results of this initial validation study indicate that the NCB is a reliable set of assessments, measuring key cognitive domains as largely accepted by the neuropsychological field. The examination of temporal reliability yielded test-retest reliability correlations which exceed the standard psychometry threshold of 0.7 for acceptable temporal reliability [17]. The NCB also demonstrated favorable temporal stability by traditional standards given the ICC values were greater than 0.5 [15].

### Limitations

The primary aim of developing the NCB has been to provide an instrument capable of reliably and remotely assessing cognition. We have sought to imbue the NCB with characteristics that facilitate the evaluation of cognitive change in group studies, as well as at the individual level. Such an approach has been advocated for some time [18] and emphasis has been placed on the need for the use of reliable measures [12]. Although the sample was diverse, the limitations of this study are the small group sample sizes, the mean age of the participants, and the use of convenience sampling to obtain participants, which impacts the generalizability of the results. All but one assessment sample met the recommended sample size of at least 30 participants [15]; nonetheless, the use of larger sample sizes and older sample populations, as well as probability sampling methods in future validation studies is warranted.

### Conclusion

This study has demonstrated that the NCB can be successfully delivered reliably and remotely, with promising clinical implications. As evidenced by the COVID-19 pandemic, there is a critical need for feasible and valid assessments for remote testing. As it has been well established that the lack of access to cognitive assessments can result in delayed diagnoses, less effective treatment, and missed lifestyle and other health-related interventions [19], the NCB addresses this critical gap as a brief, reliable, and easy-to-administer assessment battery. Additional research regarding psychometric properties, usability, and feasibility is currently underway to determine the magnitude of the clinical validity of the NCB as part of a clinician’s diagnosis process.

## Abbreviations

CANTAB: Cambridge Neuropsychological Test Automated Battery
DSST: Digit Symbol Substitution
ICC: intraclass correlation coefficient
Kendall τ: Kendall rank correlation coefficient
MTurk: Amazon Mechanical Turk
NCB: Neurotrack Cognitive Battery
s_w_: within-subject standard deviation
